# Long non-coding RNA discovery across the genus *anopheles* reveals conserved secondary structures within and beyond the Gambiae complex

**DOI:** 10.1186/s12864-015-1507-3

**Published:** 2015-04-23

**Authors:** Adam M Jenkins, Robert M Waterhouse, Marc AT Muskavitch

**Affiliations:** Boston College, Chestnut Hill, MA 02467 USA; Department of Genetic Medicine and Development, University of Geneva Medical School, rue Michel-Servet 1, 1211 Geneva, Switzerland; Swiss Institute of Bioinformatics, rue Michel-Servet 1, 1211 Geneva, Switzerland; Computer Science and Artificial Intelligence Laboratory, Massachusetts Institute of Technology, 32 Vassar Street, Cambridge, MA 02139 USA; The Broad Institute of MIT and Harvard, 415 Main Street, Cambridge, MA 02142 USA; Biogen Idec, 14 Cambridge Center, Cambridge, MA 02142 USA

**Keywords:** Anopheles, Malaria, lncRNA

## Abstract

**Background:**

Long non-coding RNAs (lncRNAs) have been defined as mRNA-like transcripts longer than 200 nucleotides that lack significant protein-coding potential, and many of them constitute scaffolds for ribonucleoprotein complexes with critical roles in epigenetic regulation. Various lncRNAs have been implicated in the modulation of chromatin structure, transcriptional and post-transcriptional gene regulation, and regulation of genomic stability in mammals, *Caenorhabditis elegans*, and *Drosophila melanogaster*. The purpose of this study is to identify the lncRNA landscape in the malaria vector *An. gambiae* and assess the evolutionary conservation of lncRNAs and their secondary structures across the *Anopheles* genus.

**Results:**

Using deep RNA sequencing of multiple *Anopheles gambiae* life stages, we have identified 2,949 lncRNAs and more than 300 previously unannotated putative protein-coding genes. The lncRNAs exhibit differential expression profiles across life stages and adult genders. We find that across the genus *Anopheles*, lncRNAs display much lower sequence conservation than protein-coding genes. Additionally, we find that lncRNA secondary structure is highly conserved within the Gambiae complex, but diverges rapidly across the rest of the genus *Anopheles*.

**Conclusions:**

This study offers one of the first lncRNA secondary structure analyses in vector insects. Our description of lncRNAs in *An. gambiae* offers the most comprehensive genome-wide insights to date into lncRNAs in this vector mosquito, and defines a set of potential targets for the development of vector-based interventions that may further curb the human malaria burden in disease-endemic countries.

**Electronic supplementary material:**

The online version of this article (doi:10.1186/s12864-015-1507-3) contains supplementary material, which is available to authorized users.

## Background

Sequencing the genome of the African malaria mosquito, *Anopheles gambiae* [1], has fueled many large- and small-scale investigations of the biology of this important vector, in an effort to develop more effective interventions to limit its harmful impacts on human health [2]. Functional genomic studies using microarrays have described basic biological processes and stimulus-responsive gene expression by detailing transcriptome profiling during the *An. gambiae* life cycle, in specific tissues, across Zeitgeber time, following blood feeding and infection, and coincident with insecticide resistance [3-11]. More recent RNA sequencing (RNAseq) studies in *An. gambiae* have described odorant receptor expression in various contexts [12,13] and other RNAseq efforts in vector insects have enabled generation of the first *de novo* transcriptome for *Anopheles funestus* [14]. Because they are designed based on existing genome annotations, gene expression microarrays cannot facilitate the discovery of unannotated genes. RNAseq is not constrained in this way, but high read depths are required for significant increases in analytical sensitivity. Most previous RNAseq studies have focused on using reads as a measure of expression of previously annotated genes, rather than discovering new genes, including new classes of genes such as lncRNAs [15-17]. Indeed, recent RNAseq of the *An. gambiae* midgut transcriptome demonstrated that high-depth sequencing can uncover many novel intergenic transcripts, including putative lncRNAs [18].

Large-scale functional genomic projects such as ENCODE and modENCODE, as well as high-throughput genomic screens, have revealed the presence of extensive sets of lncRNAs in humans (approximately 9,300), as well as in model organisms (e.g., approximately 900 in nematodes and 1,100 in fruit flies) [19-27]. The functions of these lncRNAs, however, remain largely unknown, with a few exceptions that include lncRNAs with defined roles in embryogenesis, development, dosage compensation and sleep behavior [27-32]. Part of the difficulty in deciphering the functionality of lncRNAs lies in their rapid evolution and the consequent reduction in levels of primary sequence conservation for lncRNAs among different organisms [33-35]. While this divergence presents some challenges, the lack of conservation could be exploited in species-specific targeted therapeutics. Indeed, it has been proposed that lncRNAs could be used as targets to regulate gene expression and development, as an alternative to the standard model of using small molecule drugs as antagonists of mRNA-encoded proteins [36]. This premise may also be extended to controlling vector-transmitted infectious diseases by identifying and perturbing non-coding RNA (ncRNA) targets in vector insects [37].

Previously successful vector control methods have begun to wane in efficacy with the development of singly and multiply insecticide-resistant mosquitoes in disease-endemic regions (e.g., [6,7]). Future malaria vector control will have to rely on new approaches, some of which may become apparent only as we develop a more complete understanding of the repertoire of mosquito coding and non-coding genes [18,37,38]. Using RNAseq across multiple mosquito life stages and both genders, our study has developed the most comprehensive deep RNAseq data set for *An. gambiae* to date, encompassing more than 500 million alignable sequence reads. Differential gene expression analysis confirms the roles of different classes of annotated protein-coding genes during key developmental phases, and quantification of protein-coding potential of previously unannotated transcripts identifies 318 new protein-coding genes and 2,949 putative lncRNAs. We find that the lncRNA gene set exhibits much lower sequence conservation across anophelines, when compared with either previously annotated protein-coding genes or protein-coding genes discovered in our study. While these lncRNA genes exhibit low sequence conservation, we provide evidence that the secondary structural features for many lncRNAs have been conserved. These newly identified lncRNAs provide a basis for an expanded understanding of lncRNAs in dipterans, and for future studies of ncRNAs within the genus *Anopheles*.

## Results

### Alignment and validation of RNAseq data sets

**Figure 1:**
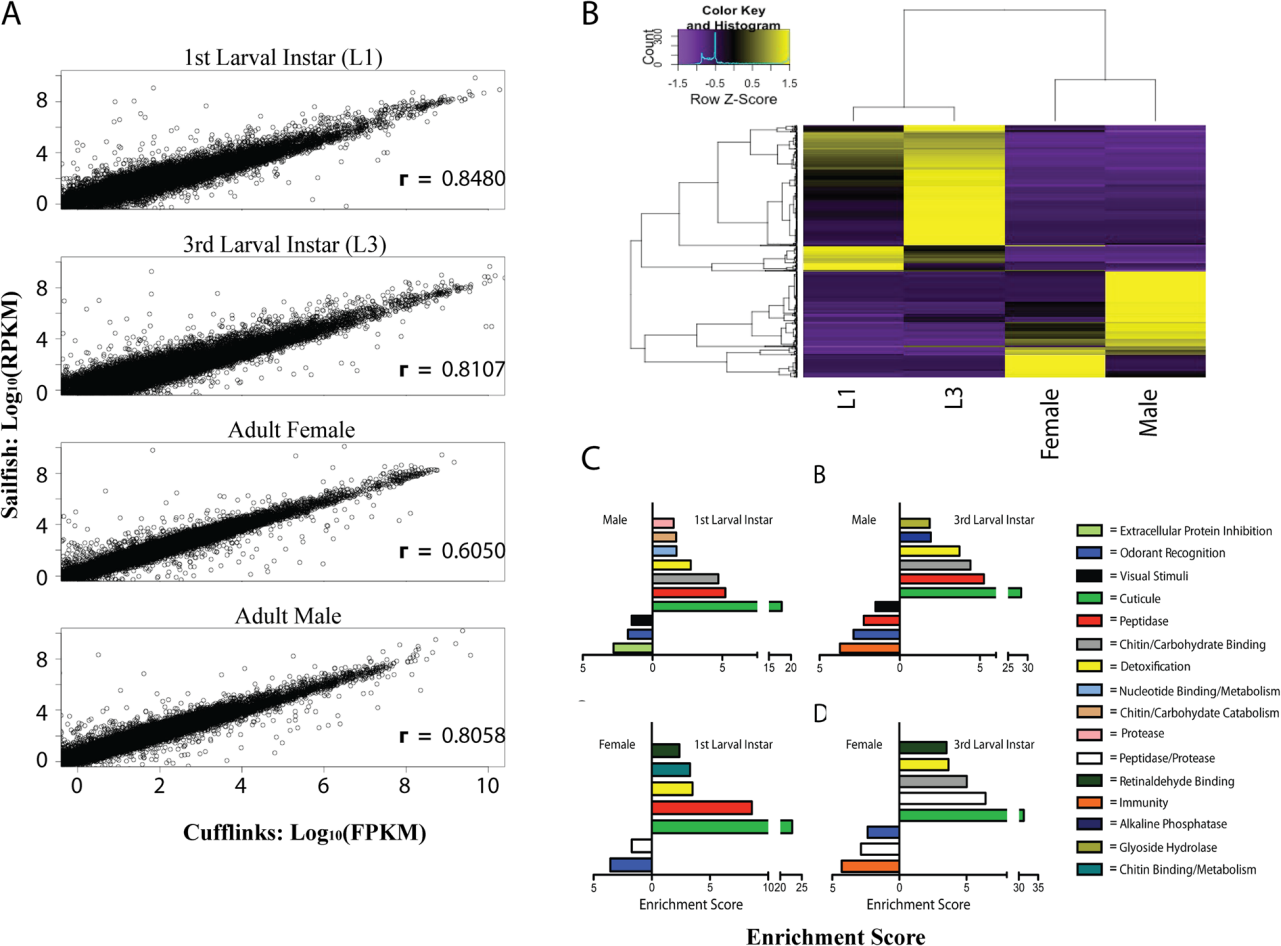
Validation of RNA-Seq library and analysis techniques. **A**. Life stage comparison of Cufflinks FPKM values to Sailfish RPKM values. Pearson’s correlation coefficient is represented for each life stage comparison. Genes used for comparison are those annotated in VectorBase release Agam3.7. **B**. Clustered FPKM expression (Additional file 1) of differentially expressed genes between life stages in *An. gambiae*. Rows and columns were clustered using Pearson correlation method with complete linkage distances. **C**. DAVID enrichment scores for differentially expressed gene groups between life stage comparisons.

Our transcriptome analysis for each life stage was supported by two RNAseq data sets: one “high read depth (HRD)” set with more than 140 million reads/stage that was used for subsequent lncRNA discovery, and one “low read depth (LRD)” set that contained approximately 30 million reads/stage that constituted biological replicates for the validation of our HRD data sets. In total, over 500 million HRD reads and over 100 million LRD reads were aligned to the *An. gambiae* PEST genome assembly AgamP3 (Table 1, see Methods). First, Cufflinks fragments per-kilobase of exonic length per million base pairs mapped (FPKM) expression values were validated against SailFish, an alignment-free quantification method that uses K-mers and defines expression levels based on reads per-kilobase of exonic length per million base pairs mapped (RPKM) [39]. The average FPKM and RPKM values between the two biological replicates produced by Cufflinks and Sailfish show Pearson correlation coefficients that were all above 0.6 (Figure 1A), indicating a high level of confidence that Cufflinks FPKM values are comparable to other, reference-free quantification methods. Using Cufflinks FPKM values, the number of differentially expressed (DE) genes identified varies greatly depending on the life stages compared, as shown by the clustered FPKM values in Figure 1B (Additional file 1). Concordant with physiological changes, fewer DE genes were identified between similar life stages, i.e., between larval stages [first larval instar (L1) and third larval instar (L3)] or between adult genders, than between larval and adult stages.

**Table 1:** **Read alignment of RNA-Sequencing data sets**

**Data set**	**Raw read count**	**Percentage mapped**	**Aligned read count**
HRD 1^st^ Instar	184,145,330	81.2%	149,517,068
HRD 3^rd^ Instar	143,507,360	76.7%	110,094,659
HRD Female	184,150,422	75.6%	139,217,446
HRD Male	194,179,892	76.8%	149,210,510
LRD 1^st^ Instar	32,425,540	79.8%	25,888,403
LRD 3^rd^ Instar	38,489,668	81.2%	31,269,540
LRD Female	27,877,821	86.7%	24,160,317
LRD Male	31,876,060	82.1%	26,162,196

Only three protein-coding genes (*AGAP007089*, *AGAP010068*, *AGAP010708*) exhibit significant decreases in expression in L3 compared to L1, while 61 are significantly up-regulated. In an adult male to adult female comparison, 44 protein-coding genes are down-regulated, while 88 are up-regulated. Adult to larval comparisons range between 133 genes up-regulated between females and L3s, the lowest such difference observed, and up to 388 genes down-regulated between males and L3s, the greatest such difference observed. When these DE genes are grouped based on their GO_Slim2 categories [40], a total of 30 major categories are identified, each of which constitutes greater than two percent of the total gene count for a given comparison (Additional file 2: Figure S2). Those categories with greater than 2 percent of the gene count are distributed across all life stage and gender comparisons. Any category that is present in less than two percent of the total DE genes for the given comparison is grouped into the “Less Than 2 percent” category; this category is the largest group for many of our comparisons. Due to the expansive nature of these categories, the DE genes were analyzed for functional enrichment using DAVID [41] to define biologically relevant groups that are differentially expressed.

Across the adult to larval comparisons, 16 categories possess an enrichment score greater than 1.5 (Figure 1C, Additional file 2: Figure S2). Genes associated with cuticle, peptidase activity, chitin/carbohydrate binding and detoxification are enriched during larval stages, when compared to adults. Genes associated with odorant recognition, immunity and visual stimuli are enriched in adults, when compared to larval stages. Overall, differentially expressed genes and their associated DAVID-enriched terms (Additional file 3) are congruent with past studies of *An. gambiae* [4,5].

### De novo identification of transcripts

**Figure 2:**
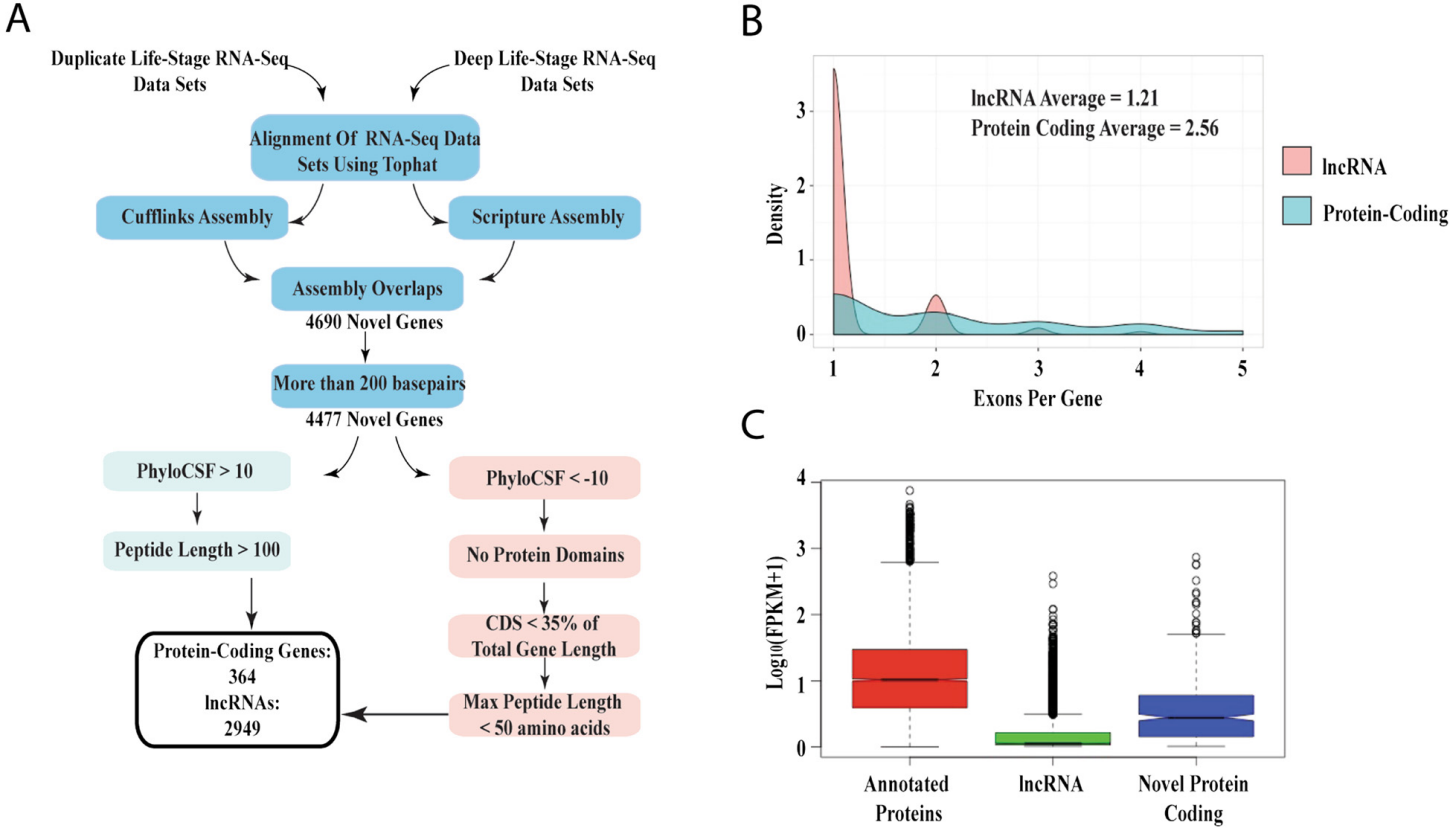
Flow chart of lncRNA and potential coding gene identification and expression/exonic structure of defined gene classes. **A**. Flow chart of lncRNA and novel protein-coding gene identification. RNAseq data sets were merged and used to produce a transcriptome that was supported by both Cufflinks and Scripture. Length, PhyloCSF score, maximum peptide length, protein domain and total coding-sequence length were used to set inclusion and exclusion criteria for the sets of lncRNAs and putative protein-coding RNAs, among the previously unannotated transcripts. **B**. Density plot of exons per-gene for lncRNAs (blue) and novel protein-coding RNAs (red). **C**. Expression values [Log_10_ (FPKM + 1)] calculated by Cufflinks for previously annotated genes in VectorBase (red), lncRNAs (green), and newly identified putative protein-coding RNAs (blue) for all genes that had an FPKM greater than zero for the merged RNAseq data set.

Cufflinks and Scripture were utilized to produce a reference annotation-based transcript (RABT) assembly – using a merged data set of all HRD RNAseq data sets – in order to identify previously unannotated RNA transcripts (Figure 2A). As the aim of this study was not to identify potential isoforms of previously annotated transcripts, only gene classes of I, U and X (intronic transcript, intergenic transcript, and exonic overlap on opposite strand, respectively) as identified by Cufflinks, were analyzed. A total of 4,690 transcripts possessed assembled transcript support by both Cufflinks and Scripture (Figure 2A). After implementing a length cutoff of 200 nt, a set of 4,477 potential transcribed loci was identified. All genes were given the identifier “Merged” (e.g., Merged.1023), based on the use of merged HRD life stage RNAseq data sets to enable the annotations.

**Figure 3:**
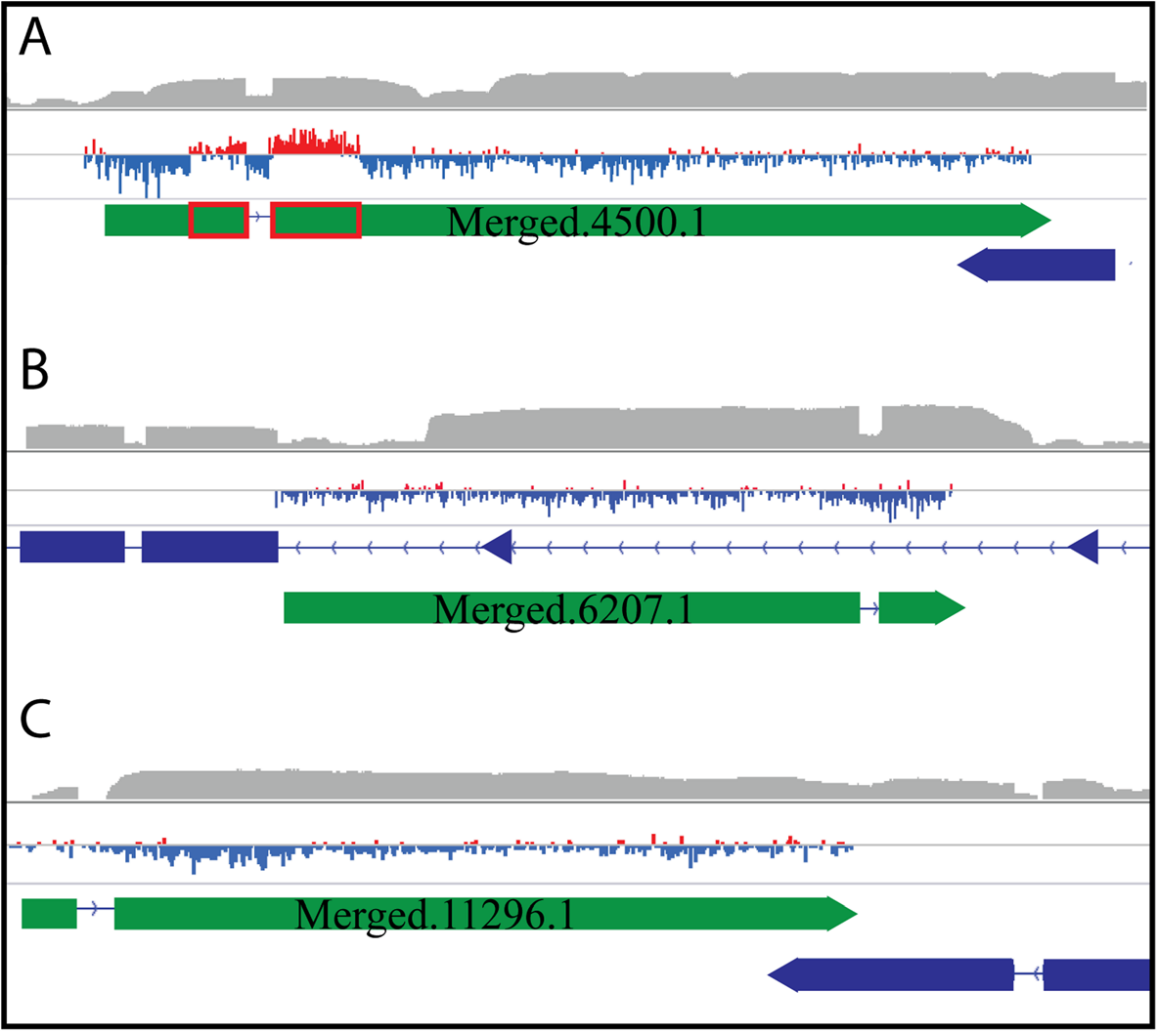
Examples of newly annotated protein-coding and lncRNA genes. Read count profiles of RNAseq alignments to a selected set of newly annotated genes, viewed using IGV (Broad Institute, Cambridge, MA) [86,87]. Chromosomal coordinate scales vary among panels. AGAP designations are given for genes encoding mRNAs (blue boxes for exons) that are complementary to newly annotated antisense lncRNAs (green boxes for exons). Strandedness of lncRNAs is determined by Cufflinks and based on output GTF file (Additional files 4 and 8). Each panel consists of the top graph indicating read depth (Log scale maximum of 6) with a PhyloCSF track below (scale −70 to 50, red indicating values above 0 and blue indicating values below 0), followed by the gene GTF track. Colored triangles indicate the orientation of the given gene. **A**. Putative protein-coding gene Merged.4500.1 maps antisense to the 3’ untranslated region of protein-coding gene AGAP007209. Regions with red boxes of Merged.4500.1 indicate the protein-coding segments of the gene (107 amino acids in length). **B**. lncRNA Merged.6207.1 maps intronically with respect to AGAP002451. **C**. lncRNA Merged.11296.1 is antisense and overlapping to AGAP011074.

Potential protein-coding mRNAs and lncRNAs were identified based on sequence and amino acid lengths, percent coding sequence and protein-coding potential (using PhyloCSF), as described in MATERIALS and METHODS. This yielded 318 potential protein-coding transcripts (Additional files 4, 5, 6, 7 and 8) and 2,949 potential lncRNAs (Additional files 4, 9 and 10). Among the 2,949 putative lncRNAs we have identified, most are intergenic transcripts (2059 lncRNAs) (Cufflinks class code “U”), while 108 are in an anti-sense orientation with respect to an exonic region of an overlapping, protein-coding mRNA (Cufflinks class code “X”), and 782 map within an intron of a protein-coding gene (Cufflinks class code “I”) (Additional file 11). For transcripts consisting of a single exon, it may be difficult for Cufflinks to predict the correct strandedness of transcript, and the pipeline may generate complementary-strand duplicate gene calls by calling the inferred transcript twice, on each of the complementary strands to which RNAseq reads align. To determine the number of genes that may have been defined as such complementary-strand duplicates we compared all genes identified and found that only 241 genes (i.e., less than 10%) exhibited 50% total overlap (Additional file 12). This implies that only a very small proportion of the transcripts identified may constitute complementary-strand duplicates rather than single gene calls. Potential protein-coding genes possess an average of 2.6 exons/gene (Figure 2B), while the lncRNA genes have, on average, 1.2 exons/gene. To further characterize the organization of the newly-annotated genes, respective FPKM expression levels were analyzed (Figure 2C). The FPKM values for the newly annotated protein-coding genes we have identified tend to be lower than those for previously identified protein-coding genes in the reference AgamP3.7 gene set, while newly identified lncRNAs tend to have mean/median FPKM values lower than those for newly annotated protein-coding genes (Figure 2C) (Additional file 13). Figure 3 illustrates examples of a novel protein-coding gene (Figure 3A), an intronic lncRNA (Figure 3B) and an anti-sense lncRNA (Figure 3C) and an intronic lncRNA (Figure 3C) that were identified in our study. Of the 2,949 lncRNA genes, 39 exhibit significant differences in expression patterns (Additional file 2: Figure S3) among life stages (Additional file 14). Comparison of our lncRNA gene set to that recently described based on a gut transcriptome [18] identifies 209 genes that possess at least 50 percent overlap (“Merged” lncRNAs exhibiting overlap can be found in Additional file 15).

### Evolutionary conservation of lncRNA sequences and secondary structures

**Figure 4:**
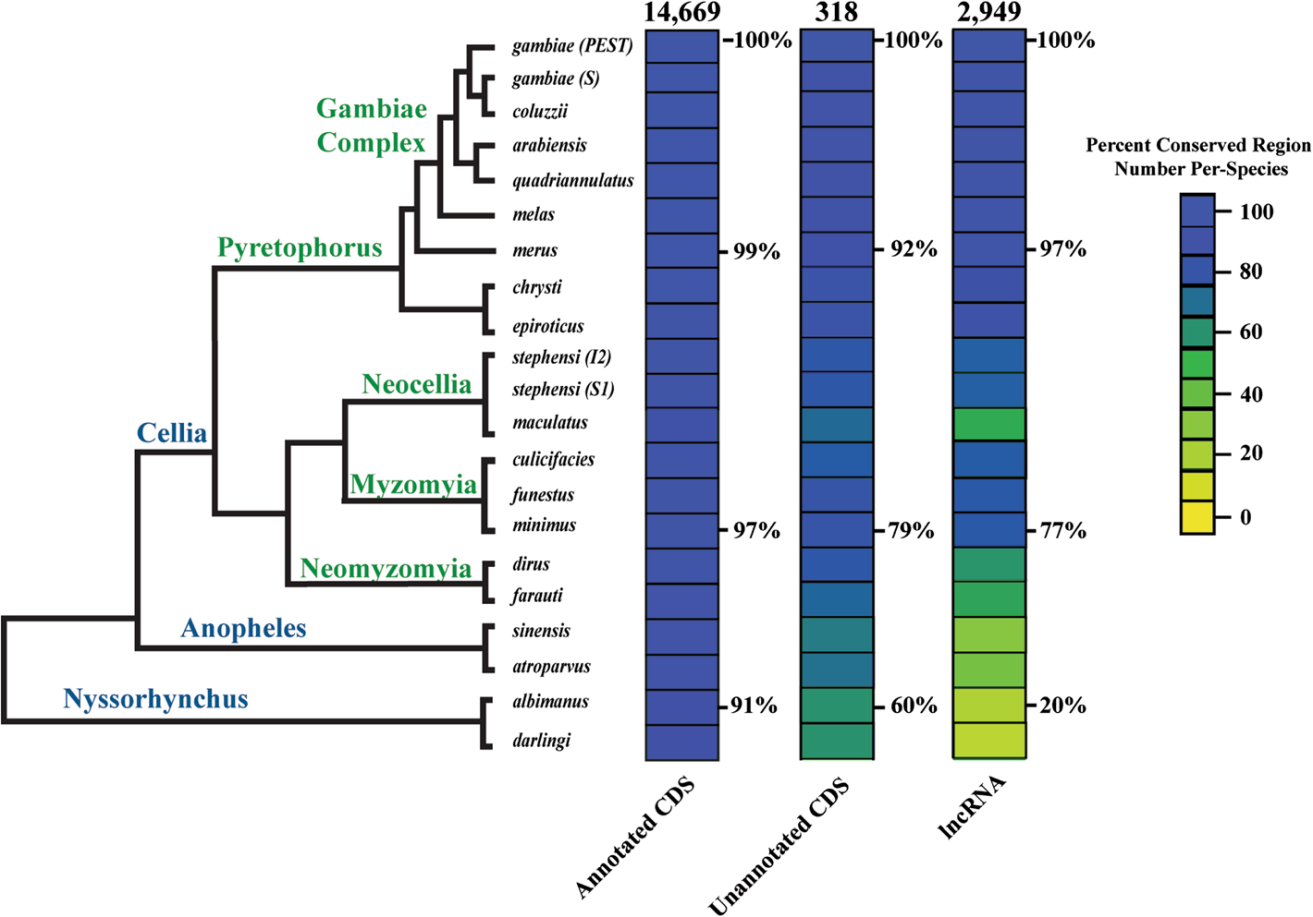
Evolutionary conservation across the genus anopheles. Percentage of previously annotated protein-coding genes (left column), newly annotated protein-coding genes (this study, middle column) and newly annotated lncRNAs (this study, right column) that could be aligned among *An. gambiae* and other comparator species using whole genome alignments. Percentages represent percent of total gene class that could be aligned to the genome of each species (heatmap colors are depicted in legend). Number of models for each class of gene, for *An. gambiae*, listed at the top of each column.

In light of recent studies of the evolutionary conservation, and the lack thereof, among lncRNAs in tetrapods [33,35], we examined the conservation of *An. gambiae* lncRNAs across the *Anopheles* genus. First, we quantified the presence/absence of lncRNA-homologous genomic regions in whole genome multiple sequence alignments across the *Anopheles* phylogeny, based on the presence/absence of an alignable region in our whole genome alignments (WGA) (Figure 4, Additional file 16: Table S1 and S2). Of the lncRNAs we have identified in *An. gambiae*, almost all exhibit conserved homologous regions within the genomes of the closely-related species within the Gambiae complex, e.g. approximately 97 percent are found in the genome of *Anopheles merus* (Figure 4). At this close evolutionary distance, similarly high percentages of homologous regions are found for the previously annotated protein-coding genes (99 percent) and the newly annotated protein-coding genes (92 percent). In the more distantly-related species, *Anopheles minimus*, of the Myzomia Series, the percentages of protein-coding genes with identifiable homologs drop to 97 percent (previously annotated) and 79 percent (newly annotated), respectively. In the most distantly related species, *Anopheles albimanus*, from the Nysorrhynchus Series, these percentages decline even further to 91 percent and 60 percent, respectively, for previously and newly annotated protein-coding genes (Figure 4). Strikingly, while 77 percent of the *An. gambiae* lncRNAs detect identifiable homologous regions in *An. minimus*, the number of conserved lncRNA-homologous regions drops dramatically, to only 20 percent, in the distant species *An. albimanus*.

**Figure 5:**
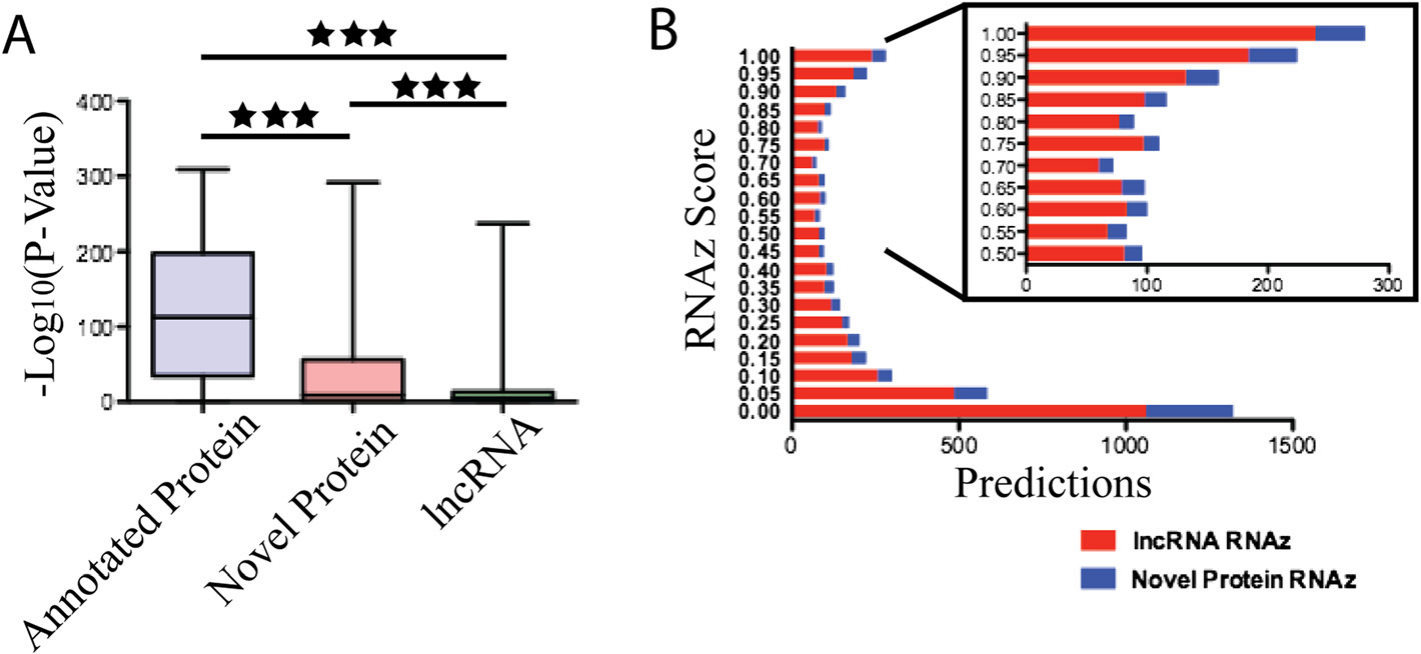
Sequence, structural and expression profiles of identified gene classes. **A**. Characterization of sequence conservation across the genus *Anopheles* performed using PhyloP. The –log_10_(PhyloP Conservation P-Value) was calculated for each gene within each respective gene class and statistical significance was determined using a Mann–Whitney *T*-Test. Starred bars denote p-value <0.001. **B**. Stacked histogram of RNAz score output from REAPR analysis (delta value of 10) for lncRNA (red bars) and novel protein-coding genes (blue bars). Insert shows confident RNA secondary structure calls with an RNAz score above 0.5.

To further characterize the conservation of lncRNAs, PhyloP was utilized to determine per-nucleotide conservation p-values across all of the genus members studied (Figure 5A). Previously annotated genes in *An. gambiae* possess higher –log(p-value of conservation) scores compared to both newly identified protein-coding and lncRNA gene classes identified in this study. The previously annotated protein-coding genes exhibit a mean (95 percent CI) value of 122.0 (120.1-123.8), newly identified protein-coding RNAs exhibit a value of 38.34 (31.88-44.80) and lncRNAs exhibit a value of 10.64 (9.958-11.32). All pairwise comparisons of the extent of conservation between all classes were significantly different (Mann–Whitney Test, p-value < 0.001).

**Figure 6:**
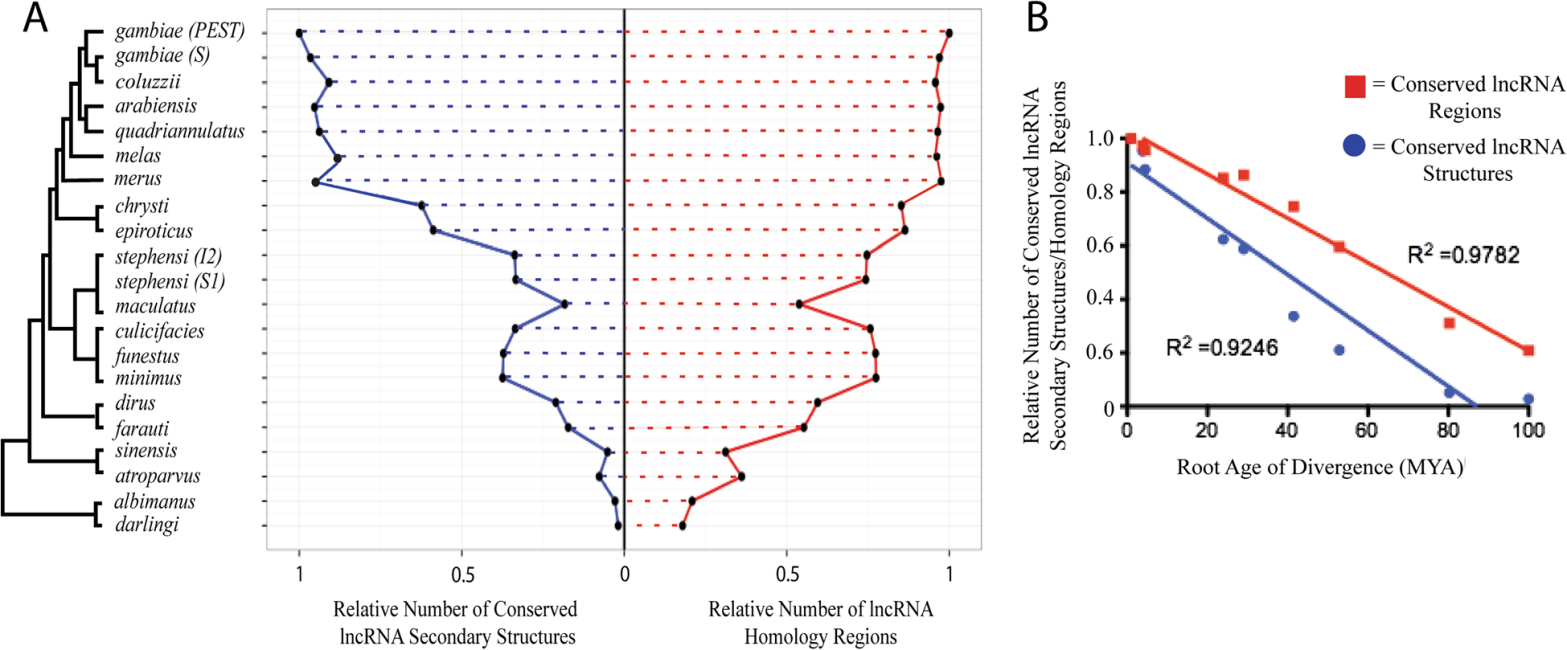
Conservation of lncRNA secondary structure and genomic regions across the *Anopheles* genus. **A**. The number of lncRNA secondary structures and conserved genomic regions per-species that are present within members of the *Anopheles* genus in relation to *An. gambiae*. Plots represent RNA sequences that possess high confidence (RNAz score > 0.5) secondary structures as identified during REAPR analysis (left, blue line) and conserved genomic regions of the lncRNA gene set (right, red line). For each species in the lineage (phylogenetic tree indicates species), the relative width of the plot corresponds to the number of confident RNA secondary structures or number of conserved genomic regions that were predicted. **B**. Change in the number of conserved genomic regions (red) and secondary lncRNA structures (blue) over time. Root age of divergence times were determined by Neafsey *et al.* [66] and lines represent linear regression. Differences in slopes between the linear regression lines are not significant based upon an ANCOVA test (P-value = 0.09).

Next, we employed REAPR (**rea**lignment for **p**rediction of structural non-coding **R**NA) to examine the conservation of RNA secondary structures in our set of newly identified transcripts. The lncRNA class contains 1,166 conserved secondary structures that possess high-confidence RNA secondary structures according to their RNAz scores (an RNAz score above 0.5 was regarded as a basis for high confidence), distributed among 835 distinct lncRNAs (Figure 5B, Additional file 2: Figure S4 and S5, Additional file 16: Table S3). By comparison, our set of newly annotated protein-coding genes contains 223 conserved RNA secondary structures among 126 distinct genes. Among the high-confidence secondary structure loci identified among lncRNAs in this study, we next analyzed the conservation of these structures across the genus *Anopheles* (Figure 6, Additional file 2: Figure S6). The genomes of species studied from the Gambiae complex exhibit high numbers of conserved secondary structures, with most genomes retaining similar numbers of conserved structures (Figure 6). Those species outside of the Gambiae complex exhibit much lower numbers of conserved secondary structures compared to *An. gambiae*, especially those species outside of the Pyretophorous Series. The 293 lncRNAs that map to genomic intervals that exhibit primary sequence conservation across all of the anopheline genomes that we analyzed possess 164 distinct secondary structural features. Those features were present in all species within the Gambiae complex, within 129 of the secondary structures we define (Additional file 2: Figure S7). Additionally, only two of the secondary structures were present in all 21 genomes analyzed. Overall, the rate of divergence for conserved secondary structures is much greater than for the conserved lncRNA-homologous genomic regions, though the observed difference is not statistically significant (p-value = 0.09) (Figure 6B.)

## Discussion

Our deep RNA sequencing has facilitated comprehensive transcriptional profiling across four *An. gambiae* life stages, identified multiple previously unannotated protein-coding genes and created the most comprehensive catalog of lncRNAs in any mosquito species, to date. Our quantification of reads mapped to genome assemblies has enabled determination of differential expression among life stages, and our aggregate data set of such genes includes many genes that have been defined as being differentially expressed in previous microarray-based studies of *An. gambiae* gene expression [4,5]. First, we compared two quantification methods, Cufflinks and Sailfish, to determine whether an alignment-free quantification method was comparable to Cufflinks and potentially preferable to currently used alignment-based methods (Figure 1A). Overall, both Cufflinks FPKM and Sailfish RPKM values are comparable and exhibit correlation values 0.6 or higher (Figure 1A). We note that we were unable to produce correlation values between Cufflinks and SailFish that were reported previously when comparing the accuracies of both methods to synthetic and qPCR data sets [39]. Combined with downstream analyses and visualization packages, we chose to use Cufflinks and its component packages for our lncRNA analysis.

Our differential gene expression profiles (Figure 1B, Additional file 1) were compared to earlier microarray-based studies to validate our RNAseq data sets. These microarray-based studies identified greater numbers of differentially expressed genes in larval-adult comparisons than in larval-larval or adult-adult comparisons, a trend of differences that is also clearly observed based on our RNA sequencing approach (Additional file 2: Figure S2). Studies by Koutsos *et al.* [4] and Harker *et al.* [5] both identified more differentially expressed genes, especially in the L1-L3 comparisons, which can be attributed to the greater number of replicates performed in their microarray studies. Similar to the Koutsos *et al.* [4] study, we identify more DE genes between males and larvae than between females and larvae. Functional classes of differentially expressed genes include many cuticular, peptidase and chitin-binding genes that are up-regulated during larval stages, and odorant recognition and immune class genes that are up-regulated in adults (Figure 1C, Additional file 3). Similar life stage-related expression patterns have been observed for immunity genes in the pollen beetle, *Meligethes aeneus* [42]. Harker *et al.* [5] described similar larval up-regulation of various gene ensembles in their study of *An. gambiae* using microarrays, including the cuticular gene A*GAP010469* and peptidase-associated genes *AGAP005671*, *AGAP001250*, *AGAP006676* and *AGAP006677*. Koutsos *et al.* [4] found genes that contain immune-related domains and fall within the pheromone-sensing GO class are up-regulated in adults, and our RNAseq-based analyses have identified similar expression patterns. The consistencies we observe in differential gene expression patterns between life stages, and in functional classes up-regulated during larval and adult life stages, respectively, engender confidence in the quality of our data set.

While approaches for the alignment of RNAseq reads to genomes are relatively mature, the task of grouping such aligned reads into lncRNAs or other gene classes remains challenging and is less well-defined. Previous classifications of lncRNAs have been based on their lengths, protein-coding potential, and maximum ORF size, and the probability of identifying full-length lncRNA transcripts using RNAseq [21,26,27,43,44]. In our study, no FPKM cutoff was utilized, as many lncRNAs have been shown to exhibit very low expression levels [35]. Implementation of our lncRNA detection pipeline (Figure 2A) identifies 2,949 lncRNAs and 318 protein-coding genes (Additional files 4 and 9). The number of lncRNAs we identify in *An. gambiae* is more than double the number identified in *D. melanogaster* and other members of the genus *Drosophila*, for which more than 1,000 long intergenic non-coding RNAs (lincRNAs) have been identified in each species, and many fewer than have been defined in studies of mice and humans, which have identified many thousands of potential lncRNAs [44,45]. As only lincRNAs have been highly studied in *D. melanogaster,* the total number of lncRNAs may be comparable in *An. gambiae*. Additionally, our putative set of lncRNA genes is smaller than that recently described for the gut transcriptome of *An. gambiae* [18]. One of the major reasons for this difference in identified lncRNAs between the two studies is that Padron *et al.* (2014) did not use a peptide length cutoff, and their protein-coding potential analyses did not take advantage of whole genome alignments. By utilizing our peptide length cutoff on their lncRNA data set and only using Cufflinks codes ‘I’,’U’, and’X’, the number of lncRNAs identified from their data set is reduced by 62 percent, to 3,740 lncRNA. Among these, only 209 genes exhibit at least 50 percent sequence overlap between the two studies. This limited overlap indicates that tissue-specific RNAseq analysis can yield a vastly different lncRNA population compared with whole organism RNAseq, which will be an important consideration for the eventual identification of a complete lncRNA gene set in *An. gambiae* and other vector insects.

Members of the lncRNA and putative protein-coding gene classes identified in our study have lower average FPKM levels and lower DNA sequence conservation, in general, than those observed for previously annotated *An. gambiae* protein-coding genes (Figure 2C). This trend of lower observed levels of expression and sequence conservation may explain why genome annotation pipelines have previously missed the putative protein-coding genes that we have defined. In addition, the average number of exons per lncRNA is much lower than the average number of exons per novel protein-coding gene that we have identified in this study (Figure 2B). This is similar to the trend in exon number per transcript that has been characterized for human lncRNAs, which have been shown to possess significantly fewer exons per gene compared to protein-coding genes [45].

Previous studies of lncRNA sequence evolution have indicated that primary sequence conservation is very low across tetrapods [33], while only a few such studies have considered conservation of secondary structure in assessing net evolutionary conservation of lncRNAs [46,47]. Those studies that have considered secondary structure have focused mainly on comparisons between a few species and not on comparisons across complete lineages, such as is now possible within the *Anopheles* genus [34,46,47]. The ability of RNA to maintain secondary structural features and associated RNA-protein interactions, even in the absence of primary sequence conservation [33,34], may underlie, in part, the increased rate of divergence for lncRNAs that has been observed in these previous studies.

Our study illustrates that across the sequenced genomes within the genus *Anopheles*, 91 percent of previously annotated protein-coding genes in *An. gambiae* exhibit matching genomic regions in *An. albimanus* (Figure 4). This level of conservation we observe is lower for the set of protein-coding genes we have newly annotated, e.g., 79 percent for *An. minimus* and 60 percent for *An. albimanus*. It is even lower for the lncRNA class, e.g., 77 percent for *An. minimus* and 20 percent for *An. albimanu*s. Furthermore, examining sequence conservation within these genomic regions using PhyloP p-values of conservation scores indicates that lncRNA sequences are much more divergent across the *Anopheles* genus, compared with previously and newly annotated protein-coding classes (Figure 5A). The reduced numbers of identifiable conserved lncRNA-homologous genomic regions is in agreement with previous findings in tetrapods, which illustrated a rapid decrease in 1:1 orthologous lncRNA families across many classes of tetrapods [33]. The proportions of lncRNAs that identify homologous genomic regions in our whole genome alignments are similar to the proportions of conserved protein-coding genes, when considering only the closely-related species within the Gambiae complex (Figure 4). However, beyond the Pyretophorus Series, the proportions of conserved lncRNA-homologous regions decline much more rapidly than those for protein-coding genes. Those putative lncRNA-harboring genomic regions that are identifiable in other species also show much higher levels of sequence divergence compared with protein-coding genes. Together, these results imply that anopheline lncRNAs diverge at a much higher rate than protein-coding genes. Accordingly, some *An. gambiae* lncRNAs present in the most recent common ancestor of the Pyretophorous Series and the Neocellia and Myzomyia Series, for example, may have diverged beyond recognition within the Neocellia and Myzomyia, while other *An. gambiae* lncRNAs may have arisen relatively recently and are therefore restricted to species within the Gambiae complex.

To extend our analysis beyond primary sequence conservation for lncRNAs, we employed REAPR to identify lncRNA secondary structures and analyze their conservation across the anophelines (Figure 6, Additional file 2: Figure S6). Among all putative *An. gambiae* lncRNAs we define, only 28 percent exhibit high-confidence RNA secondary structures. Although it has been proposed that all lncRNAs should possess a functional secondary structure, this premise has not been validated at the genome-wide level for other sets of related organisms, nor has the conservation of lncRNA secondary structures across multiple related species in other clades been analyzed and described in comparable depth [32,48-50]. The closely related members of the Gambiae species complex, in which homologous genomic regions are found for almost all *An. gambiae* lncRNAs, all exhibit similar proportions of high-confidence RNA secondary structures within these lncRNAs. While these structures are highly conserved within the Gambiae species complex, the numbers of lncRNA secondary structures conserved relative to *An. gambiae* decline rapidly for species outside of the complex, at an apparent rate even more pronounced than the decline in the numbers of conserved lncRNA-homologous genomic regions (Figure 6A). However, when corrected for the root age of divergence for each species analyzed, we see that primary sequences and secondary structures exhibit similar rates of divergence (Figure 6B). Both of these rates are much higher than those that have been described for lncRNAs in chordates [33]. Increased divergence rates in insects, as compared to chordates, have been noted previously for protein-coding genes [51,52]. Rapid divergence of lncRNA sequences as compared to protein-coding genes (Figures 4 and 6) has also been reported for rodent species [34].

These differences in the number of conserved lncRNA regions and number of secondary structures across the anophelines, especially evident for those lncRNAs that exhibit conserved genomic regions in all species but secondary structures in only a subset of those species (Additional file 2: Figure S7), imply that lncRNA secondary structures tend to evolve after a most recent common ancestor for a given set of species has acquired transcriptional activation of particular genomic loci. This finding is consistent with the long-acknowledged idea of “pervasive transcription” across the genome [53]. Pervasive transcription describes the process by which most regions of the genome are transcribed, including those that fail to encode proteins or functional ncRNAs. Through random mutations, these “pervasive” transcripts acquire protein-coding ability or a functional RNA structure, over evolutionary time. Selective pressure causes these altered transcripts to become fixed within a population if they are advantageous for the organism. Given the evolutionary interval between the onset of transcriptional activation of a particular genomic region and the time at which the transcript becomes functionally beneficial, some lineages/species that have evolved during that time period may express a particular pervasive transcript before it becomes a functionally beneficial transcript within that species or lineage.

Increased evolutionary rates of lncRNA sequences compared to protein-coding genes may contribute to bionomic diversity that has been observed across the genus *Anopheles* by affecting the evolution of species-specific behaviors, such as resting, mating and feeding patterns [54,55], just as behavioral control has begun to be attributed to variation among *Drosophila* lncRNAs [28]. The notion that lncRNAs modulate the activities of protein-coding genes is well-established [17,56,57]. However, we speculate that lncRNA-mediated regulation of gene expression, coupled with the rapid evolution of lineage-specific lncRNA ensembles in mosquitos, may underlie the rapid diversification of vector mosquito behaviors [58] for which it has been, thus far, difficult to define differentiating causal mechanisms. Our deep RNA sequencing of *An. gambiae* has provided the most comprehensive catalog of lncRNAs in mosquitoes to date, and presents the prospect of identifying a new generation of targets for approaches to vector control that will enable further reductions in the burden of human malaria.

## Conclusion

Malaria is a life-threatening infectious disease for which half of the global human population is at risk. Malaria control currently relies on population control of vector mosquitos of the genus *Anopheles*. Vector control methods are becoming less effective due to the propagation of insecticide resistance alleles within and between many *Anopheles* populations. In order to identify new prospective targets for vector control, we have identified a pan-genomic set of almost 3,000 long non-coding RNAs (lncRNAs) in *Anopheles gambiae*, the predominant vector of human malaria in sub-Saharan Africa. Members of this lncRNA set evolve much faster across the *Anopheles* genus than do protein-coding genes, but they retain conserved secondary RNA structural features across the genus. In contrast, we find that lncRNA sequences and secondary structure are highly conserved among six species within the Gambiae Complex. Continuing analysis of these lncRNAs will provide new insights in vector biology that can be applied to develop next-generation vector control methods.

## Methods

### Colony and sequencing

*Anopheles gambiae* G3 colony (courtesy of Dr. Flaminia Catterucia, Harvard School of Public Health, Boston, MA, USA) was reared with an 11:11 Light:Dark (L:D) photoperiod with a one-hour crepuscular period between light and dark stages. Adults were fed 10 percent glucose solution *ad libitum*, and both genders were kept in the same cage. First larval instar (L1) and third larval instar (L3) stages were removed from the colony within 12 hours of emergence from chorion or previous larval cuticle, respectively. Adults were sampled three days post-emergence, and all samples were collected at approximately eight hours into the light cycle of the 11:11 LD photoperiod. All samples were kept in RNA-Later (Ambion, Austin, TX) until RNA extraction and sequencing. The L1 and L3 life stages were chosen because they represent early and late stages during larval development, which can be synchronized clearly, and because previous studies have defined a set of contigs that are differentially expressed between these stages [4]. Future lncRNA discovery studies may include the pupal stage, due to its importance for the completion of morphogenesis that yields the adult mosquito.

High read depth (HRD) paired-end RNA sequencing was performed at the Broad Institute (Cambridge, MA) using a Qiagen RNAeasy Mini Kit for RNA extraction and the Illumina TruSeq RNA Sample Preparation Kit v2, and libraries were sequenced on the HiSeq 2000 platform. Low read depth (LRD) paired-end RNA sequencing of larval replicates was performed by Otogenetics Corp. (Atlanta, GA), using the same protocol as the HRD samples. Low read depth adult single-end RNA sequencing data sets were obtained from Pitts *et al.* (2011). All RNA sequencing data produced have been submitted to the European Nucleotide Archive and can be accessed under the SRA Accession number of PRJEB5712.

### RNAseq read alignment and analysis

HRD RNAseq reads were soft clipped, and replicate RNAseq reads from Otogenetics Corp. were subsequently hard clipped by 10 bp on both the 5’ and 3’ ends of each read (Additional file 2: Figure S1). First, hard clipping of the LRD replicate samples was performed to reduce the number of potential adapter sequences, even though read quality scores were high overall, as the reads were long enough to support such hard-clipping (~100 bp in length). Second, clipping the reads makes their length more comparable to other replicate reads from Pitts *et al.* (2011) that were trimmed as previously described. Reads were aligned to the *An. gambiae* AgamP3 genome assembly, which was softmasked using RepeatMasker (www.vectorbase.org) [59,60]. Alignment, transcriptome assembly and analyses were performed using the Tuxedo Suite [61-63], which comprises Tophat2, Cufflinks, Cuffmerge and Cuffdiff2 programs, Scripture and Sailfish [39,64]. Splice junction mapping was performed using Tophat2 (version 2.0.10) with a mismatch (−N) appropriation of 3 and a read-edit-dist of 3. Cufflinks (version 2.1.1) was run with default settings using the *An. gambiae* AgamP3.7 annotation –gtf function and a reference annotation-based transcript (RABT) assembly. Scripture (Beta-2 version) was run using default settings. Cuffmerge was used to combine and filter artifacts from the resulting transcriptome assemblies from Cufflinks, Scripture and the reference *An. gambiae* AgamP3.7 annotation. Cuffdiff2 was used to determine differentially expressed genes of interest with an FDR of 0.05 and the –u (multi-read correct) function, and differentially expressed genes were determined using the Benjamini-Hochberg correction, with two replicates for each life stage (HRD and LRD for each stage). In order to validate the FPKM (fragments per kilobase of exonic length per million reads) values produced by the Tuxedo Suite, Sailfish was used to compare values. Sailfish was run with default parameters and the average RPKM (reads per kilobase exonic length per million reads mapped) was compared to FPKM values determined using Cufflinks.

### Identification of newly annotated transcripts

HRD RNAseq data sets for all four stages and genders (L1, L3, Male, Female) were combined and aligned using Tophat2, as previously described [61]. Cufflinks and Scripture were subsequently used to identify newly annotated transcripts. Cuffcompare was used to compare newly annotated transcripts to the *An. gambiae* AgamP3.7 gene set. To identify probable lncRNAs, class codes “I”, “U” and “X” were used in Cufflinks (as this study does not aim to identify potential novel isoforms of known protein-coding genes, the “J” class was not utilized).

### *Anopheles* genome alignments and PhyloCSF scanning for protein-coding potential

A set of 21 available *Anopheles mosquito g*enome assemblies species were retrieved from VectorBase [60]. These included assemblies of *An. gambiae* PEST [1], *An. gambiae* Pimperena S form and *An. coluzzii* (formerly *An. gambiae* M form) [65], the species sequenced as part of the *Anopheles* 16 Genomes Project [66], *An. darlingi* [67], and the South Asian species *An. stephensi* [68]. Details of assemblies used can be found in Additional file 16: Table S1.

Multiple whole genome alignments of 21 available *Anopheles* assemblies were built using the MULTIZ feature of the Threaded-Blockset Aligner suite of tools [69], employing a similar approach to that used for other multi-species whole genome alignments such as those for 12 *Drosophila* [70] and 29 mammal [71] genomes. Before computing the alignments, repetitive regions within each of the input genome assemblies were masked. Assemblies were analysed using RepeatModeler [72] to produce repeat libraries that were then combined with known repeats from *An. gambiae* and retrieved from VectorBase, before being used to mask each genome assembly using RepeatMasker [59]. The 21-species maximum likelihood phylogeny, required to guide the progressive alignment approach of MULTIZ, was estimated using RAxML [73] from the concatenated protein sequences of Genewise [74] gene predictions using Benchmarking sets of Universal Single-Copy Orthologs (BUSCOs) from OrthoDB [75], and rooted with predictions from the genomes of *Aedes aegypti* [76] and *Culex quinquefaciatus* [77]. The MULTIZ approach first runs all-against-all pairwise LASTZ alignments (default settings), followed by projections ensuring that the reference species is “single-coverage,” with projection steps guided by the species dendrogram to progressively combine the alignments.

Examining patterns of evolutionary conservation across multiple whole genome alignments can help to distinguish protein-coding regions from non-protein-coding regions, e.g., as in the analyses of 12 *Drosophila* [70] and 29 mammal [71] genomes. Specifically, PhyloCSF [78] is a method developed to determine whether a multi-species nucleotide sequence alignment represents a protein-coding region, based on patterns of evolutionary conservation such as codon substitution frequencies (CSF). Thus, PhyloCSF can be used to help distinguish protein-coding and non-coding RNAs represented among new transcript models obtained from high-throughput transcriptome sequencing. Gene transfer format (GTF) files (from Cuffmerge output) defined the required genomic intervals for PhyloCSF analyses per codon, per exon, and per gene. Per-codon analysis scanned each transcript region (plus flanking 50 bp) in the six translational frames to score for protein-coding potential across the entire region. Per-exon analysis identified the best-scoring translational frame for the length of each exon, and per-gene analysis identified the best-scoring, start-codon-to-stop-codon open reading frame of the complete annotated transcript.

Coding transcripts were classified as those new transcripts that possess an open reading frame >100 amino acids in length and a PhyloCSF score greater than ten (i.e., 10 times more likely to be coding than non-coding). Non-coding transcripts were classified as those novel transcripts that possess a maximum open reading frame < 50 amino acids in length, an open-reading frame that is < 35 percent of the total transcript length, a PhyloCSF score less than negative ten, and no recognizable domains as defined by PFAM, TIGRFAM or SUPERFAMILY libraries [79-81], which were searched using HMMER with default settings for e-value cutoffs (website version 1.9) [82].

### Differential gene expression and categorization

Using the Cuffdiff function as described above, differentially expressed (DE) genes were defined using a false discovery rate of 0.05. Gene Ontology (GO) terms [83] were extracted for those DE genes from VectorBase [60]. These GO terms were grouped by GO_Slim2 categories with CateGOrizer [40]. To define the groups or classes of genes that are DE, DAVID [41] was utilized to determine enrichment scores. DE genes were compared in order to define genes that were up/down-regulated, regardless of adult gender and regardless of larval life stage.

### Determining conservation and secondary structure of newly annotated genes across *anopheles* lineages

In order to quantify the sequence conservation of the lncRNA and newly annotated protein-coding classes of genes, we employed PhyloP. First, PhyloFIT, part of the PHAST package (version 1.3) [84], was utilized to create a nonconserved substitution model from the multiple genome alignments, using four-fold degenerate sites. Using PhyloP, part of the same PHAST package, the p-value of conservation was then calculated for all genes identified in this study or for genes in the *An. gambiae* AgamP3.7 annotation release, for comparisons. For analysis, only newly annotated genes that had strandedness predicted by Cufflinks were used.

REAPR (**rea**lignment for **p**rediction of structural non-coding **R**NA) was utilized to determine secondary structure scoring of identified lncRNA class members using the RNAz score [49] . Realignment of the lncRNA genes using REAPR was performed using a delta value of 15 and the --alistat functions. For confident secondary structures, only loci possessing RNAz scores over 0.5 were used, as these correspond to an FDR of ~ 0.04 as described in RNAz 2.1 documentation [85]. Rate of degradation of number of secondary structures and conserved genomic regions was determined using a linear regression and ANCOVA test to determine significance . Analyses were performed using GraphPad Prism 5.0b for Mac, GraphPad Software, San Diego, California USA, www.graphpad.com.

### Availability of supporting data

The data sets supporting the results of this article are available in the European Nucleotide Archive, under accession PRJEB5712 (http://www.ebi.ac.uk/ena/data/view/PRJEB5712). All files produced by Scripture, PhyloP and REAPR, along with all whole genome alignment and gene alignment files, can be accessed freely at http://bioinformatics.bc.edu/~jenkinad/.

## Additional files

Additional file 1:
**Expression of** ***Anopheles gambiae*** **genes across life-stages.**
Additional file 2: Figure S1. Representative quality scores of LRD samples. **Figure S2.** GOSLIM2 terms of genes that exhibit differential expression among life stages/genders. **Figure S3.** lncRNAs that exhibit differential expression among life stages/genders. **Figure S4.** RNAz scores of secondary structures in lncRNA and novel protein coding genes after REAPR realignment. **Figure S5.** Secondary structures for a differentially expressed lncRNA. **Figure S6.** Histogram of number of genomes aligned to for high-confidence secondary structure. **Figure S7.** Clustering of conserved secondary structures in lncRNAs that are present in all *anopheles* species. Additional file 3:
**DAVID enrichment classes for each life-stage comparison.**
Additional file 4:
**GTF file produced by Cufflinks of newly annotated genes.**
Additional file 5:
**FASTA file containing coding sequence of all putative protein-coding genes identified in this study.**
Additional file 6:
**FASTA file containing full length mRNA sequence of all putative protein-coding genes identified in this study.**
Additional file 7:
**File containing putative peptides identified in this study with alignment scores and PhyloCSF scores.**
Additional file 8:
**GFF3 file of all genes identified in this study.**
Additional file 9:
**List of identifiers for lncRNA and putative-protein coding genes identified.**
Additional file 10:
**FASTA file containing sequences of all lncRNA.**
Additional file 11:
**Cufflinks class codes for all newly identified genes.**
Additional file 12:
**List of lncRNA that have 50% overlap to a gene on the complementary strand, as defined by Cufflinks.**
Additional file 13:
**Expression of all** ***Anopheles gambiae*** **genes, including newly identified genes across life-stages.**
Additional file 14:
**Differential expression analysis of all** ***Anopheles gambiae*** **genes, including newly identified genes across all life-stages.**
Additional file 15:
**GTF file of all lncRNA that exhibit 50% overlap to previously identified lncRNAs in** ***Anopheles gambiae*** **midgut.**
Additional file 16:
**Table S1.** Genomes utilized for whole genome alignments and associated *anopheles* species. **Table S2.** Number of 1:1 conserved lncRNA regions in each anopheline genome assembly. **Table S3.** Number of high-confidence lncRNA secondary structures in each anopheline genome assembly.
