# Supplementary material for: Long non-coding RNA discovery across the genus anopheles reveals conserved secondary structures within and beyond the Gambiae complex
Source: BMC Genomics. 2015 Apr 23;16(1):337. doi: 10.1186/s12864-015-1507-3 (PMC4409983; doi:10.1186/s12864-015-1507-3)
Supplement: Additional file 2: Figure S1. — Representative quality scores of LRD samples. Figure S2. GOSLIM2 terms of genes that exhibit differential expression among life stages/genders. Figure S3. lncRNAs that exhibit differential expression among life stages/genders. Figure S4. RNAz scores of secondary structures in lncRNA and novel protein coding genes after REAPR realignment. Figure S5. Secondary structures for a differentially expressed lncRNA. Figure S6. Histogram of number of genomes aligned to for high-confidence secondary structure. Figure S7. Clustering of conserved secondary structures in lncRNAs that are present in all anopheles species. [file 12864_2015_1507_MOESM2_ESM.pdf]

A.

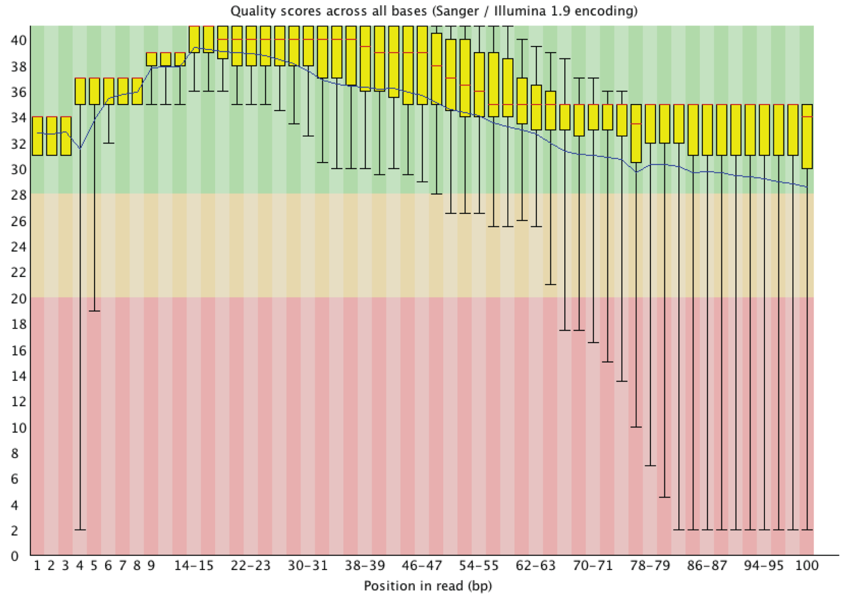

B.

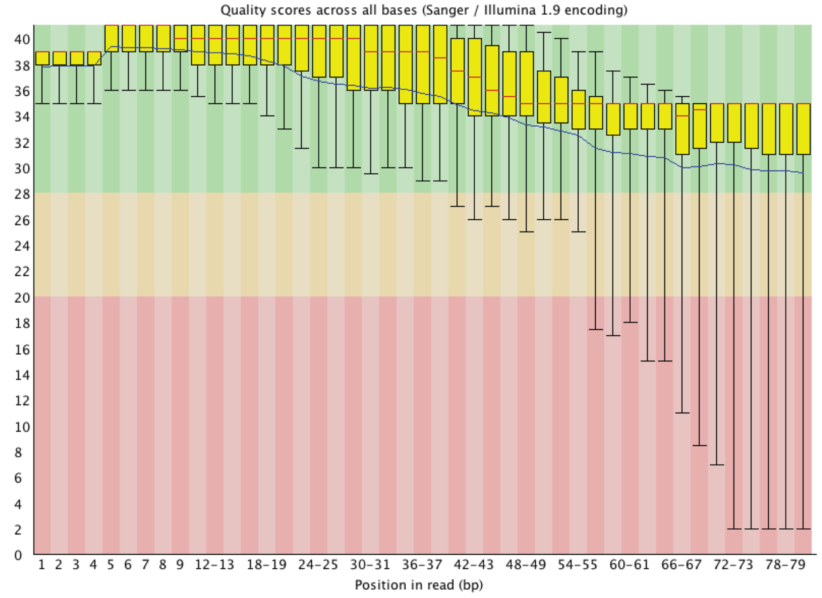

Additional Figure 1: Representative Quality Scores of LRD Samples
A. Quality scores of L1 RNAseq reads before trimming. Visualized using FASTQC (http://www.bioinformatics.bbsrc.ac.uk/projects/fastqc) **B.** Quality scores of L1 RNAseq reads after trimming 10 nucleotides from each end of the read.

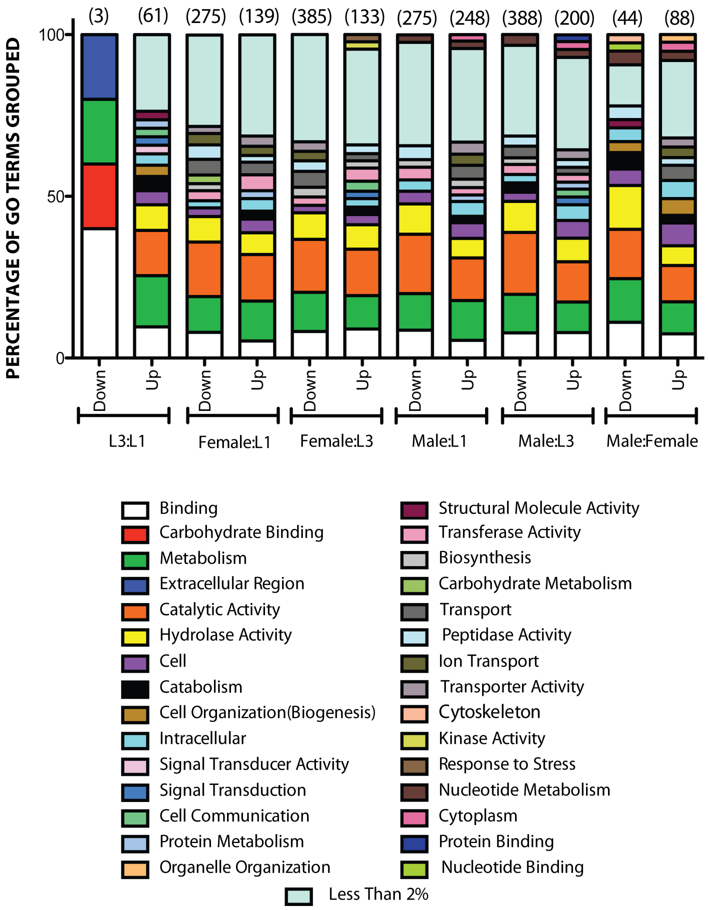

Additional Figure 2: GOSLIM2 Terms of Genes that Exhibit Differential Expression Among Life Stages/Genders

Differentially expressed genes for each pairwise life stage comparison (as indicated on the x-axis) grouped using CateGOrizer into GOSLIM2 terms [1]. Numbers at top of each group indicate number of differentially expressed genes for the comparison in either the up- or down-regulated direction. Each category is represented as the percentage of total GOSLIM2 terms grouped. The “Less Than 2%” category represents GOSLIM2 categories that represent less than 2% of the total terms grouped for a given comparison. Categories not within this group represent more than 2% of the total genes grouped for a given comparison.

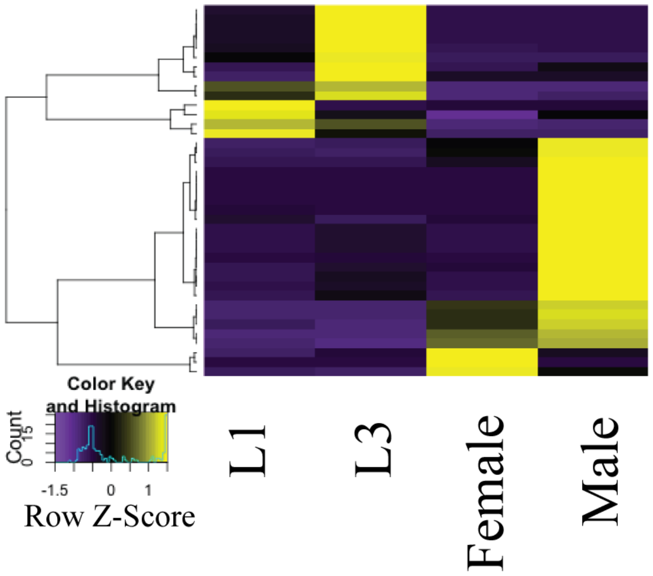

Additional Figure 3: lncRNAs that Exhibit Differential Expression Among Life Stages/Genders

Row Z-score expression (FPKM) of differentially expressed lncRNAs, as determined by Cuffdiff2 [2], between life-stages in *An. gambiae*. Rows were clustered using Pearson correlation method with complete linkage distances (see Materials and Methods)(Supp. File 7)

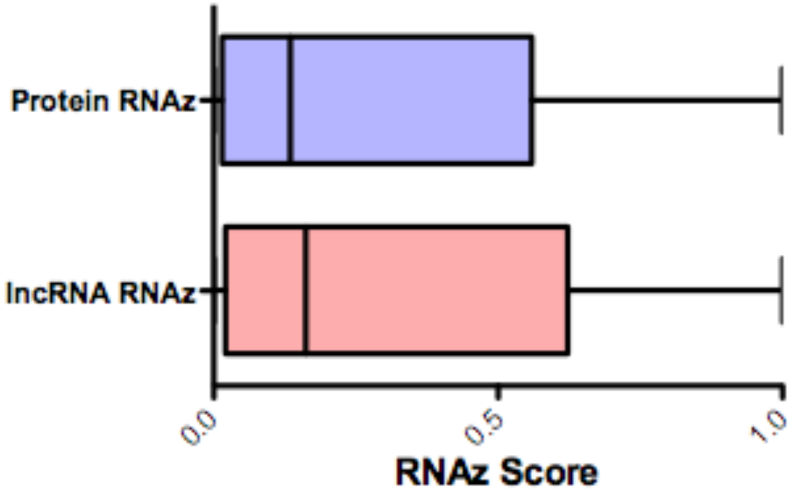

Additional Figure 4: RNAz Scores of Secondary Structures in lncRNA and Novel Protein Coding Genes After REAPR Realignment

RNAz scores for loci identified during REAPR analysis [3]. RNAz scores were calculated using a delta value of 10 for secondary structure realignment based on original whole genome alignments.

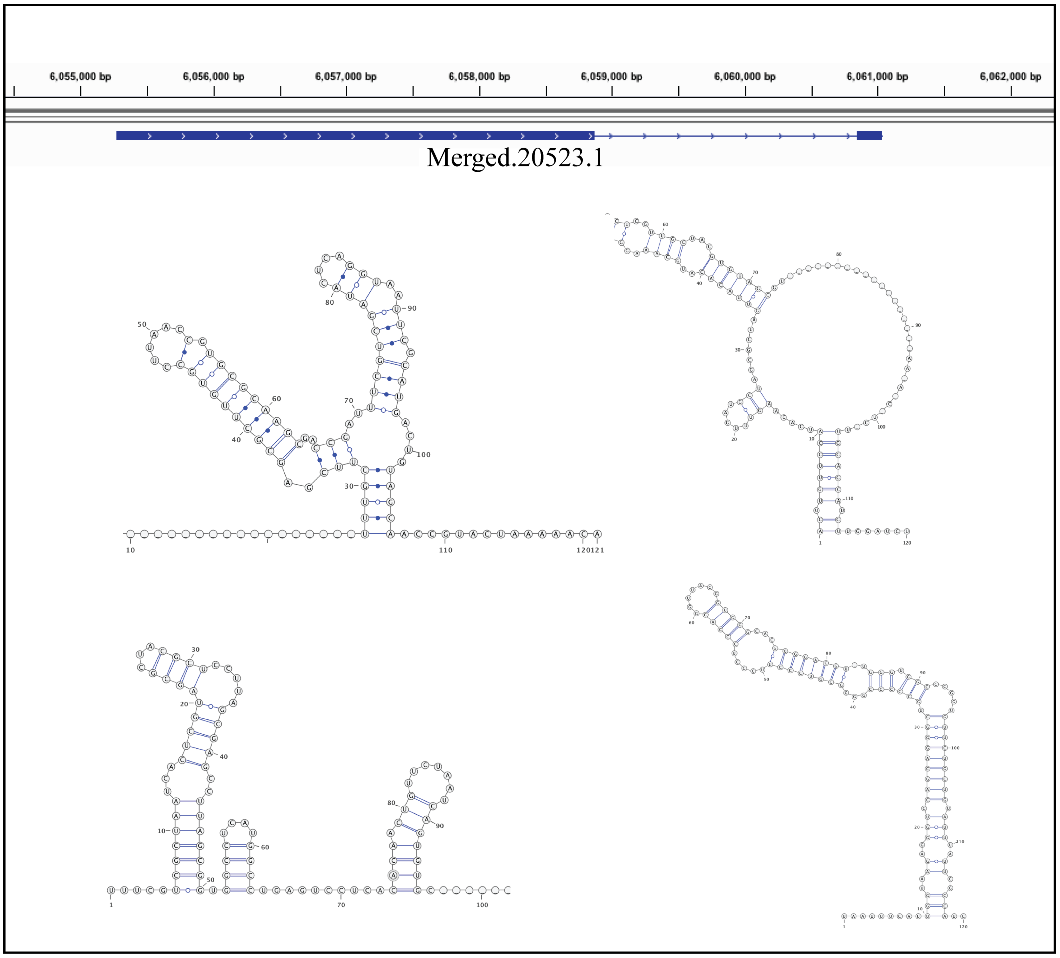

Additional Figure 5: Secondary Structures for a Differentially Expressed lncRNA
Differentially expressed lncRNA Merged.20523.1 is shown with the gene structure and coordinates on the X-chromosome (visualized using IGV) [4,5]. REAPR analyses reveal multiple high confidence secondary structure loci within the gene, four of which are depicted. RNA secondary structures were visualized using VARNA [6].

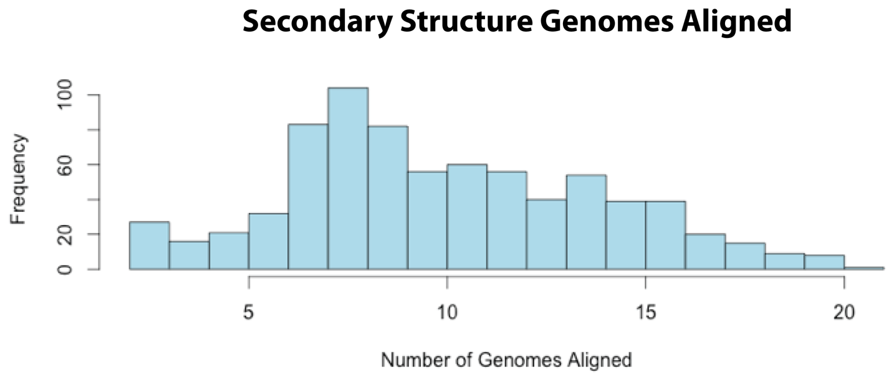

Additional Figure 6: Histogram of Number of Genomes Aligned to For High-Confidence Secondary Structure

Distribution of the numbers of genomes aligned for each stable RNA secondary structure locus identified during REAPR analysis. REAPR analyses were performed using a delta value of 10, and a high-confidence secondary structure cutoff was placed at a value of 0.5, as described in previous RNAz publications [7].

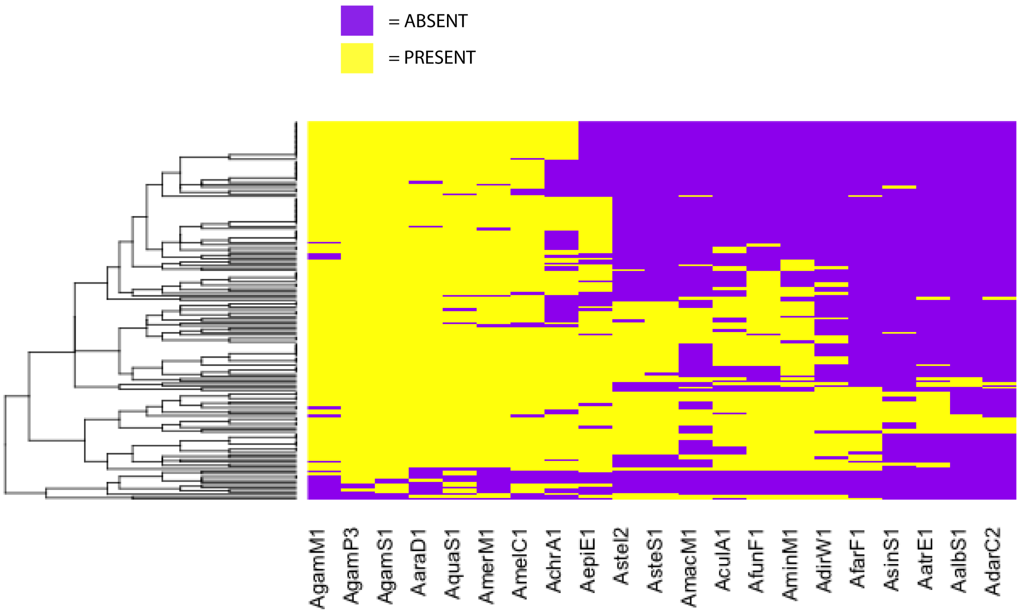

Additional Figure 7: Clustering of Conserved Secondary Structures in lncRNAs that are Present in All *Anopheles* Species: Of the 293 lncRNAs for which we identify conserved genomic regions in the genome assemblies analyzed, a subset of 90 include a total of 164 distinct secondary structures. These 164 structures were clustered based on presence (yellow) or absence (purple) in each assembly. Each structure was clustered using Pearson correlation method with complete linkage distances. Dendrogram on y-axis indicates the hierarchical clustering relationships. Genome names on x-axis correlate to the species name listed in Supplemental Table 1.

REFERENCES

1. Hu Z-L, Bao J, Reecy J (2008) CateGOrizer: A Web-Based Program to Batch Analyze Gene Ontology Classification Categories. OJB 9: 108–112.

2. Trapnell C, Hendrickson DG, Sauvageau M, Goff L, Rinn JL, et al. (2013) Differential analysis of gene regulation at transcript resolution with RNA-seq. Nat Biotechnol 31: 46–53. doi:10.1038/nbt.2450.

3. Will S, Yu M, Berger B (2013) Structure-based whole-genome realignment reveals many novel noncoding RNAs. Genome Res 23: 1018–1027. doi:10.1101/gr.137091.111.

4. Thorvaldsdóttir H, Robinson JT, Mesirov JP (2013) Integrative Genomics Viewer (IGV): high-performance genomics data visualization and exploration. Brief Bioinform 14: 178–192. doi:10.1093/bib/bbs017.

5. Robinson JT, Thorvaldsdóttir H, Winckler W, Guttman M, Lander ES, et al. (2011) Integrative genomics viewer. Nat Biotechnol 29: 24–26. doi:10.1038/nbt0111-24.

6. Blin G, Denise A, Dulucq S, Herrbach C, Touzet H (2009) Alignments of RNA structures. IEEE/ACM Trans Comput Biol Bioinform 7: 309–322. doi:10.1109/TCBB.2008.28.

7. Gruber A., Findeiß S, Washietl S, Hofacker I., Stadlet PF (2010) RNAz 2.0: improved noncoding RNA detection. Pacific Symp Biocomput: 69–79.
