# Supplementary material for: Long non-coding RNA discovery across the genus anopheles reveals conserved secondary structures within and beyond the Gambiae complex
Source: BMC Genomics. 2015 Apr 23;16(1):337. doi: 10.1186/s12864-015-1507-3 (PMC4409983; doi:10.1186/s12864-015-1507-3)
Supplement: Additional file 16: — Table S1. Genomes utilized for whole genome alignments and associated anopheles species. Table S2. Number of 1:1 conserved lncRNA regions in each anopheline genome assembly. Table S3. Number of high-confidence lncRNA secondary structures in each anopheline genome assembly. [file 12864_2015_1507_MOESM16_ESM.pdf]

Species	Assembly
Anopheles gambiae PEST	AgamP3
Anopheles gambiae Pimperena S form	AgamS1
Anopheles coluzzii Mali-NIH M form	AgamM1
Anopheles merus	AmerM1
Anopheles arabiensis	AaraD1
Anopheles quadriannulatus A	AquaS1
Anopheles melas	AmelC1
Anopheles chrysti	AchrA1
Anopheles epiroticus	AepiE1
Anopheles minimus A	AminM1
Anopheles culicifacies A	AculA1
Anopheles funestus	AfunF1
Anopheles stephensi	AsteS1
Anopheles stephensi	AsteI2
Anopheles maculatus B	AmacM1
Anopheles farauti	AfarF1
Anopheles dirus A	AdirW1
Anopheles sinensis	AsinS1
Anopheles atroparvus	AatrE1
Anopheles darlingi	AdarC2
Anopheles albimanus	AalbS1

Additional Table 1: Genomes Utilized for Whole Genome Alignments and Associated *Anopheles* Species

Species' Genome	Number of 1:1 lncRNA
gambiae (PEST)	2949
gambiae (S-Form)	2729
gambiae (M-Form)	2694
arabiensis	2739
quadriannulatus	2714
merus	2743
melas	2691
christyi	2398
epiroticus	2431
stephensi (I2)	2101
stephensi (S1)	2091
maculatus	1515
culicifacies	2130
minimus	2179
funestus	2176
dirus	1675
farauti	1555
atroparvus	1017
sinensis	877
albimanus	588
darlingi	505

Additional Table 2: Number of 1:1 Conserved lncRNA Regions in Each Anopheline Genome Assembly: All number of conserved regions are based upon LASTZ identification during WGA alignment.

Species' Genome	Number of Secondary Structures
gambiae (PEST)	1129
gambiae (S1)	1091
gambiae (M1)	1027
arabiensis	1077
quadriannulatus	1060
merus	1072
melas	1000
christyi	704
epiroticus	664
stephensi (I2)	381
stephensi (S1)	377
maculatus	207
culicifacies	379
minimus	423
funestus	420
dirus	238
farauti	195
atroparvus	87
sinensis	58
albimanus	32
darlingi	21

Additional Table 3: Number of High-Confidence lncRNA Secondary Structures in Each Anopheline Genome Assembly: Number of high-confidence lncRNA (RNAz Score > 0.50) secondary structures identified in each *Anopheles* genome.
